# Research highlights, *The Plant Genome*, Volume 16, Issue 4

**DOI:** 10.1002/tpg2.20420

**Published:** 2023-12-20

## 1 EPIGENOME AND EPITRANSCRIPTOME IN PLANT–ENVIRONMENT INTERACTIONS

Understanding how plants react and adapt to different environmental conditions is essential for crop improvement in sustainable agriculture. Recent technological advances have enabled high‐throughput profiling of plant epigenome and epi‐transcriptome, which are important aspects in gene regulation in plant response to environmental signals. While numerous studies about epigenetic regulations have been conducted in model plants, there is a need to integrate the knowledge and put more effort into exploring the mechanisms for improving agronomic traits in crops. Yung et al. (https://doi.org/10.1002/tpg2.20404) summarize the highlights of articles in the special issue “Epigenome and Epitranscriptome in Plant–Environment Interactions,” which provides new insights into the theoretical basis and technical considerations in investigating epigenetic regulations in different crop species.

## 2 LEGUME EPIGENETICS UNDER ABIOTIC STRESSES

Understanding the epigenetic regulations of abiotic stress responses is important for improving legume production. Yung et al. (https://doi.org/10.1002/tpg2.20237) reviewed the current findings on the roles of epigenetic features, including DNA methylation, histone modifications, and noncoding RNAs, in abiotic stress responses in legumes. In recent years, next‐generation sequencing technologies have facilitated the investigation of epigenomic changes. The relationships between the changes in the global levels of epigenetic modifications and stress tolerance varied among legume species, suggesting that analyses of the specific modified genes and the pathways being represented in the omics data are necessary. Integrative analyses involving multiple epigenetic features are needed for a better understanding of their interaction in transcriptional regulation. The inheritability of epigenetic modifications should also be assessed in future studies to achieve epigenetic optimization of gene expressions across generations.

## 3 TRANS‐KINGDOM RNAs AND PLANT–ENVIRONMENT INTERACTIONS

RNAs can travel from one organism to another and function in interacting organisms. Increasing studies have reported that noncoding RNAs can be exported from plants to fungal pathogens, parasitic plants, insects, and nematodes. Noncoding RNAs may be sorted in plant cells according to their sequence motifs, terminal nucleotides, and modifications and are secreted by extracellular vesicles, RNA‐binding proteins, or other secretion pathways. These extracellular RNAs are sequentially absorbed by interacting organisms to modulate cognate gene expression, and this process can be antagonized by the proteins secreted from interacting organisms. Recent advances in this field were discussed in this review by Liu et al. (https://doi.org/10.1002/tpg2.20289).

## 4 CURRENT TECHNIQUES FOR PLANT EPITRANSCRIPTOMICS

The epitranscriptome is well recognized as an additional regulatory layer in plant growth and development. Xie et al. (https://doi.org/10.1002/tpg2.20316) review epitranscriptomic modifications found so far in plants and the recent development and application of technical advancements in plant epitranscriptomic studies. The usage and application of third‐generation sequencing technologies are discussed in detail with case studies. RNA modifications, including chemical modifications, RNA editing, and transcript isoforms, could lead to systemic changes in gene expressions in response to environmental fluctuations. This review highlights the importance of epitranscriptomics in the study of gene regulatory networks in plants and encourages multi‐omics investigations using recent technological advancements.

## 5 EPIGENETICS REGULATES PHOTOPERIODIC FLOWERING

In response to changeable season, plants precisely control the initiation of flowering in appropriate time of the year to ensure reproductive success. The change in day length (photoperiod) caused by seasonal changes is an important environmental cue to regulate flowering in various plants. Epigenetics is well known for altering gene function without changing the DNA sequence. In this review, Liu et al. (https://doi.org/10.1002/tpg2.20320) summarize recent works about how epigenetics regulates the photoperiod‐mediated flowering pathway, discuss their importance in this biological process, and give a brief prospect for future study directions.

## 6 ALMOND MODELS PLANT EPIGENETICS

While genomes were originally seen as static entities, recent advances in genomics have revealed that they interact with internal and external factors through epigenetics. Nowhere is this relationship more complex and poorly understood than in the plant kingdom, particularly in perennial woody crops, where the unique biology of plants intersects with human and environmental factors. Fresnedo Ramírez et al. (https://doi.org/10.1002/tpg2.20367) used almond as a model of the scientific advances and phenomena surrounding plant epigenetics. Starting with an explanation of the current model of a dynamic genome, epigenetic mechanisms in plants are addressed through their effects on aspects such as control of post‐winter dormancy, self‐incompatibility in pollination, and disorders affecting canopies. These examples provide insight into the role epigenetics could have in the future of crop breeding, disease management, and resilience to climate change.

## 7 ALLERGEN‐DEPLETED FOOD CROPS

Foodborne allergies are a serious health concern globally, as few of these pose life‐threatening consequences. Vadhtya et al. (https://doi.org/10.1002/tpg2.20375) bring insights on different aspects of food allergy problems and find prospects for developing food crops with reduced allergen content. The solution lies in the efforts of human science researchers in developing tolerance since childhood as well as plant science researchers in developing food crops with reduced allergen proteins. The emerging technologies and methods provide hope for developing food crops with reduced allergen content but not allergen‐free. This article also suggests documenting and making available data on crop‐specific allergen problems across the world to understand the exact scale of adverse impact on human health, economics, and society.

## 8 MINERAL NUTRIENTS AND ABIOTIC STRESS

Global climate is changing more rapidly than ever, threatening plant growth and productivity while exerting considerable direct and indirect effects on the quality and quantity of plant nutrients. In this context, Khan et al. (https://doi.org/10.1002/tpg2.20362) discuss the molecular mechanisms related to the readjustment of nutrient pools for sustained plant growth under harsh conditions and channeling the minerals to edible sources (seeds) to address the “One Health” strategy for maintaining plant health and productivity. The recent advancement of CRISPR/Cas9‐based genome editing technologies in the development of climate‐resilient crops with enhanced nutrient content and optimized yield may also be ensuring food and nutrition security.

## 9 DISCOVERY OF GENES REGULATING GROUNDNUT SEED WEIGHT

Seed weight in groundnut has direct impact on yield as well as market price because of the preference of consumers and industry for bold seeds. Gangurde et al. (https://doi.org/10.1002/tpg2.20265) used a quantitative trait locus‐sequencing (QTL‐Seq) approach to identify the genomic regions associated with seed size in groundnut using a recombinant inbred line (RIL) population. QTL‐Seq analysis uncovered 182 single nucleotide polymorphisms (SNPs) in the genomic regions on chromosomes B06, B08, and B09. Eleven SNPs were identified as nonsynonymous in the genomic regions of 10 candidate genes. The *BIG SEEDs* locus associated with groundnut seed weight was identified in genomic region on chromosome B09. Four diagnostic kompetitive allele specific polymerase chain reaction (KASP) markers were developed and validated for deploying genomic‐assisted breeding to improve seed weight in groundnut.

## 10 BIOLOGICAL KNOWLEDGE MATTERS FOR TRAIT PREDICTION

The most abundant tocochromanols in maize grain have low vitamin E activity, thus providing an opportunity for boosting vitamin E levels. Tanaka et al. (https://doi.org/10.1002/tpg2.20276) tested several statistical models that incorporated existing biological information to improve the prediction of tocochromanol levels in maize grain. When incorporating previously known quantitative trait loci for maize grain tocochromanols in a statistical model, predictive abilities were improved by 7.0%–13.6% compared with a commonly used prediction method. The highest average improvement (15.4%) in predictive ability was achieved with a multi‐trait model that had a tocochromanol phenotype and transcript abundances in developing grain for one to three large‐effect causal genes as multiple response variables. These results illustrate the enhancement of trait prediction models when informed by prior biological knowledge, leading to a potentially more rapid development of maize cultivars with high vitamin E levels.

## 11 TRAINING POPULATION DESIGN ENABLES PREDICTION IN EXOTIC MAIZE

Plant products are the main source of vitamin E (tocochromanols), an essential nutrient in the human diet; therefore, breeding crops with higher tocochromanol content has the potential to improve human health. Exotic germplasm is an important source of the genetic diversity required for plant breeding, but it is difficult to predict using existing adapted training populations. Tibbs‐Cortes et al. (https://doi.org/10.1002/tpg2.20286) found that optimal training population design methods based on data mining techniques enabled genomic prediction of tocochromanol levels in exotic‐derived maize. These results suggest a promising approach to efficiently incorporate novel germplasm into plant breeding programs.

## 12 GWAS OF MONOSACCHARIDES IN RICE GRAIN

Monosaccharides are the building blocks for the synthesis of polymers or complex carbohydrates in plant tissues, such as rice grain. Genetic dissection of monosaccharides in rice grain was conducted using a genome‐wide association study (GWAS). Panahabadi et al. (https://doi.org/10.1002/tpg2.20292) reported 17 genomic regions (QTLs) significantly associated with monosaccharides contents; 33 genes were introduced as candidate genes, from which two transcription factors were noted.

## 13 META‐QTLS INCREASES RICE GRAIN ZN CONTENT

Zinc (Zn) malnutrition affects more than 2 billion people globally. Biofortification of rice with Zn is the most cost‐effective approach to address Zn malnutrition in Asia. Genomics‐assisted breeding can fast‐track the development of Zn‐biofortified rice. Joshi et al. (https://doi.org/10.1002/tpg2.20315) detected 57 meta‐QTLs for grain Zn that were enriched with diverse metal homeostasis genes involved in production of root exudates, metal uptake, and transport, partitioning, and loading into rice grains. These genes were differentially expressed in vegetative and reproductive tissues, and a complex web of interactions was observed among them. Superior haplotypes and their combinations were identified for nine candidate genes. The precise MQTLs with high phenotypic variance, candidate genes, and superior haplotypes help efficient Zn biofortification of rice.

## 14 GENOMIC SELECTION ACCURACY FOR MICRONUTRITIONAL TRAITS IN WHEAT

As a major source of carbohydrates and protein, wheat is consumed by ≥2.5 billion people worldwide. However, the wheat grain is deficient in micronutrients, including iron (Fe), Zn, and vitamin A. Thus, biofortification in wheat involving improvement in the concentrations of grain Fe, Zn, and vitamin A has received major attention in cereal breeding programs. It is anticipated that genomic selection, which makes use of hundreds of thousands of genetic markers, will help to enhance genetic gain to achieve the goal of biofortification. Meher et al. (https://doi.org/10.1002/tpg2.20332) assessed the genomic selection accuracy for three micronutrient traits such as grain Fe, Zn, and β‐carotenoid concentrations (a precursor for vitamin A) using eight Bayesian regression models. The Bayes Ridge Regression model was observed to achieve the highest genomic selection accuracy. The genomic prediction accuracy increased with increase in the size of the training population as well as with increase in marker density. The results of the study suggest that genomic selection can be used for improvement of the grain micronutrient contents in wheat.

## 15 IMPROVING THE AMINO ACID CONTENT OF WHEAT GRAIN

The amino acid content of wheat grain has important consequences for human health. Free (soluble, nonprotein) asparagine, for example, is converted to the probably carcinogenic processing contaminant, acrylamide, during baking, toasting, and high‐temperature processing, whereas lysine is an essential dietary amino acid for monogastric animals, including humans, and is not present in sufficient amounts in cereal grains to provide adequate nutrition. Oddy et al. (https://doi.org/10.1002/tpg2.20335) report that free amino acid concentrations in wheat grain vary widely in their heritability and in the genetic loci that control them, but that free asparagine content could be decreased to some extent and lysine concentration increased through the selection of appropriate alleles. Wheat pangenome resources were used to identify candidate genes underlying the control of lysine concentration, opening up possibilities for future attempts to increase the lysine content of wheat grain.

## 16 ENHANCING NUTRITIONAL VALUE OF RICE

White rice has a low nutritional value and a high glycemic index. This has led to an increase in type‐2 diabetes in countries where white rice is a dietary staple. Certain phenolic compounds have been shown to confer many health benefits, and some are associated with a reduction in starch digestibility. Mbanjo et al. (https://doi.org/10.1002/tpg2.20360) have identified rice genotypes with enhanced nutritional potential. Colocalization was found between nutritional traits, and nutritional and color‐related traits. Targeted association analysis identified 67 SNPs, located within 52 candidate genes, associated with 24 traits. Six haplotypes identified within the genes *Rc/bHLH17* and *OsIPT5* indicated that these genes have an important role in the regulation of a wide range of phenolic compounds, and not only those directly conferring pericarp color. These genetic linkages identified between nutritionally valuable phenolic compounds and pericarp color present not only a valuable resource for the enhancement of the nutritional value of rice but an easy method of selecting suitable genotypes.

## 17 GENOMICS OF HIGH IRON AND ZINC CONTENT

Zinc and iron deficiencies can be found throughout the world, especially in developing countries, and both are linked to poverty and malnutrition. To address this problem through the fortification of important food crops, such as groundnut, genomics‐assisted breeding can be deployed for accelerated breeding of biofortified varieties with high iron and zinc content. In this context, this study employed genetic mapping approach and unveiled five major genomic regions associated with iron content and four major genomic regions for zinc content. Interestingly, Parmar et al. (https://doi.org/10.1002/tpg2.20361) identified three co‐localized associated genomic regions for Fe and Zn content. These associated genomic regions are home to key candidate genes such as *ZIP transporter*, *bZIP transcription factor*, and *vacuolar iron transporter*, which are pivotal in regulating Fe and Zn accumulation in seeds. These findings unravel the genetic basis of groundnut's nutritional richness, paving the way for improved breeding strategies and enhanced nutritional impact. Furthermore, the identified genomic regions and candidate genes hold promise for future fine mapping efforts and the development of diagnostic markers for high Fe and Zn content in groundnut.

## 18 THE GENETICS OF DRIED DATE COLOR

Some types of fruit are commercially produced more for their dry stage than their fresh stage. This is true for dates as it is for figs, cranberries, and apricots, among others. Often times, dry fruit color is an important factor in market value, and its biology remains rarely studied. Younuskunju et al. (https://doi.org/10.1002/tpg2.20373) conducted a GWAS with dry dates and identified genomic regions associated with how light or dark a dried date is. These regions were distinct from the gene previously shown to affect red or yellow fresh date color. These findings should help begin to understand the biological changes in drying fruit.

## 19 k‐MERS UNLOCK CAUSAL VARIANT DISCOVERY

GWASs allow the discovery of associations between genetic markers and traits of agronomic interest in crops. However, such studies often fail to pinpoint causal variants or genes due to incomplete marker datasets or focus on particular types of variants. Lemay et al. (https://doi.org/10.1002/tpg2.20374) report that the occurrence of k‐mers, that is, short sequences of nucleotides obtained from whole‐genome sequencing datasets, can be used as markers to facilitate the discovery of causal variants in GWAS. This approach pinpointed known causal variants at four soybean loci and suggested promising candidate genes at other loci whose causal variants are yet unknown. The software released along with this paper will allow other researchers to benefit from these methods.

## 20 LOSS OF CULTIVATED GENETIC DIVERSITY

Genetic erosion, understood as a reduction in allelic evenness and richness, is directly related to the breeding capabilities, vulnerability, evolutionary potential, and resilience of crops. The possible loss of genetic diversity was investigated by comparing the diversity maintained in the Regional Service for Agrofood Research and Development (SERIDA) collection for 30 years with the currently cultivated material of the bean market class Fabada. Seed phenotyping and high‐throughput genotyping (GBS) were combined to analyze the genetic diversity in these materials. Jurado et al. (https://doi.org/10.1002/tpg2.20379) observed a genetic erosion in the currently cultivated material, and it cannot be attributed to the diffusion of modern cultivars. In addition, redundant lines and homonymies among the accessions classified as market‐class Fabada and preserved in the SERIDA collection were found, which will help the efficient preservation of species diversity.

## 21 MULTIPLE QTL CONDITIONING WHITE MOLD RESISTANCE

QTL identification and their use for selecting white mold (WM) resistance genotypes are crucial for accelerating dry bean breeding with improved WM resistance. Meta‐QTL WM2.2, WM5.4, and WM7.5 have major effects on WM resistance. Combining the newly developed pipeline Khufu *de novo* QTL‐seq and classic QTL mapping, Roy et al. (https://doi.org/10.1002/tpg2.20380) validated the effects of WM2.2, WM5.4, and WM7.5 QTL in two mapping populations. Our findings revealed that meta‐QTL WM2.2 consists of three separate regions; WM5.4 was delimited to 22.60–36.25 Mb in the heterochromatic regions, whereas WM7.5 was fine mapped to a 0.83 Mb genomic interval. Additionally, WM4.2 QTL was fine mapped to a 0.89 Mb interval. Strong candidate genes conferring WM resistance were found for the detected meta‐QTL genomic regions. Acquired knowledge of the narrowed major QTL intervals, flanking markers, and candidate genes provides promising opportunities to the design of functional genetic markers for genomic‐assisted selection breeding and functional validation of the candidate genes to improve WM resistance in dry bean cultivars.

## 22 BEAN BREEDING STRATEGY DICTATES GENOMIC SELECTION SUCCESS

The approach taken to implement genomic selection in a bean breeding program will impact the magnitude of population improvement and the predictive ability of the genomic prediction model. In a series of simulation experiments, Chiaravallotti et al. (https://doi.org/10.1002/tpg2.20383) report that prediction accuracy and genetic gain varied along with trait, breeding strategy, and population size. Trait genetic architecture and heritability had the greatest impact on genomic selection, so traits of interest should aid breeders in determining the ideal breeding strategy. Each breeding strategy impacts genetic variance and allele frequency differently. Therefore, depending on a breeder's goals (e.g., maintaining variance or maximizing gain in a particular trait), care should be taken in designing the breeding pipeline, constructing and updating the model training population, and genotyping the training population.

## 23 SALINITY STRESS TOLERANCE PREDICTION FOR BIOMASS‐RELATED TRAITS IN MAIZE USING GENOME‐WIDE MARKERS

Genomic prediction of complex and difficult‐to‐measure traits such as salinity tolerance may enhance genetic gain in food crops. Singh et al. (https://doi.org/10.1002/tpg2.20385) reported that marker density, individual trait, heritability, and model selection may influence the predictive ability of genomic selection for salinity tolerance in maize. A significantly higher predictive ability was observed when we used only trait‐associated markers instead of the higher‐density maker sets. Bayesian models showed higher predictive ability than genomic best linear unbiased prediction (GBLUP) for salt tolerance index of shoot length, root length, and root weight traits. For the shoot and root length stress tolerance index, epistasis in the extended GBLUP (EGBLUP) and reproducing kernel Hilbert spaces (RKHS) models resulted in a higher predictive ability than GBLUP.

## 24 MACHINE LEARNING FOR PREDICTING PERFORMANCE OF SUGARCANE CLONES

Sugarcane has a highly complex genome, with multiple species ancestry and a high level of ploidy. For key traits, like cane yield, substantial epistasis has been demonstrated. Machine learning methods for genomic prediction have been suggested to be able to capture complex interactions between genomic features. Using a large dataset of sugarcane clones with yield data and genome‐wide SNP array data, Chen et al. (https://doi.org/10.1002/tpg2.20390) found machine learning methods, including deep learning, were almost competitive with genomic best linear unbiased prediction (routinely used for genomic predictions), particularly when extended to account for dominance and epistasis.

## 25 CAMELINA WINTER HABIT CONTROLLED BY THREE COPIES OF FLC

Winter crops provide both agronomic and environmental benefits; thus, efforts are being made to adapt the annual *Camelina sativa* for use as a winter cover crop. Chaudhary et al. (https://doi.org/10.1002/tpg2.20397) utilized genetic mapping and gene expression analyses to identify three genomic regions that independently contribute to the expression of the winter phenotype. Notably, all three homologous regions contain a candidate flowering time gene, *Flowering Locus C* (*FLC*), suggesting that depending on the genetic source, each copy of *FLC* can be functional. Germplasm carrying all three active loci has yet to be identified, so manipulation of the three will facilitate the development of novel winter germplasm that can expand the acreage of this versatile alternative oilseed.

## 26 NEW LOCI FOR SOYBEAN PROTEIN AND OIL CONTENT

Soybean seed protein and oil content are important traits for human food and animal feed. Vuong et al. (https://doi.org/10.1002/tpg2.20400) detected a single major genomic region associated with these two traits and mapped it to chromosome 20 of the soybean genome. This region has not been reported before. Additional investigations were also conducted to identify candidate genes harbored in this region, leading to the characterization of gene functionality. This discovery enhanced the understanding of the roles and effects of these genes on performance of these traits and also provided valuable sources for soybean scientists to develop new soybean varieties with desirable protein and oil content.

## 27 SKIM EXOME CAPTURE EXPLOITS THE DATA TO COST RATIO

One of the most limiting factors of genetics and breeding research for crop improvement is cost. While costs for generating sequencing data continue to decrease as technology develops, the wheat genome is very large and complex, limiting what information can be gained for wheat that can be accomplished in smaller, less complex staple crops with existing genomic platforms. Jordan et al. (https://doi.org/10.1002/tpg2.20381) developed a new method called skim exome capture (sec), which combines the strengths of existing sequencing and genotyping technologies and allows for targeted sequencing of the genic regions of wheat, resulting in a more informative snapshot of the wheat genome at a reduced cost per sample compared to traditional re‐sequencing and capture approaches. This new approach is repeatable and can be scaled to a very attractive price point (<$8/sample), generating useful genotypic data for downstream breeding applications such as GWASs or genomic selection that can help produce better‐adapted wheat varieties.

## 28 NEAR‐GAPLESS GENOME ASSEMBLIES FOR TWO SOYBEAN CULTIVARS

Developing high‐quality gapless genome assemblies is pivotal for understanding the gene organization, evolution, and characterization of genes associated with traits of interest. Here, Garg et al. (https://doi.org/10.1002/tpg2.20382) report near‐gapless genome assemblies of two important soybean cultivars—Williams 82 and Lee. The misassembled regions of these genomes have been corrected by deploying the long‐read sequencing technology and optical mapping data. Precisely, 10 chromosomes in Williams 82 and eight in Lee were constructed as a single contig with no gaps, and protein‐coding genes have been reported for these genomes. The near‐gapless genomes for two cultivars and annotations will be a significant resource for genomics‐assisted crop improvement efforts in soybean.

